# Antibiotic prescribing patterns in the placement of dental implants in Europe: A systematic review of survey-based studies

**DOI:** 10.4317/medoral.26450

**Published:** 2024-04-14

**Authors:** Ángel Orión Salgado-Peralvo, Naresh Kewalramani, Alba Pérez-Jardón, Mario Pérez-Sayáns, María Victoria Mateos-Moreno, Lorenzo Arriba-Fuente

**Affiliations:** 1 ORCID: 0000-0002-6534-2816. DDS, MSc, MPH, PhD. Department of Dental Clinical Specialties, Faculty of Dentistry, Complutense University of Madrid, Madrid, Spain; 2 ORCID: 0000-0002-5162-2841. DDS, MSc. Department of Nursery and Stomatology, Rey Juan Carlos University, Madrid, Spain; 3 ORCID: 0000-0001-5174-8922. DDS, MSc. Oral Medicine, Oral Surgery and Implantology Unit (MedOralRes), Faculty of Medicine and Dentistry, University of Santiago de Compostela, Spain. Health Research Institute of Santiago de Compostela (IDIS), ORALRES Group, Santiago de Compostela, Spain; 4 ORCID: 0000-0003-2196-9868. DDS, MDS, FDS, PhD. Oral Medicine, Oral Surgery and Implantology Unit (MedOralRes), Faculty of Medicine and Dentistry, University of Santiago de Compostela, Spain. Health Research Institute of Santiago de Compostela (IDIS), ORALRES Group, Santiago de Compostela, Spain. Instituto de los materiales de Santiago de Compostela (iMATUS), Spain; 5 ORCID: 0000-0002-8237-4596. DDS, MSc, PhD. Department of Dental Clinical Specialties, Faculty of Dentistry, Complutense University of Madrid, Madrid, Spain; 6 ORCID: 0000-0002-6029-8887. MD, MSc, PhD. Department of Dental Clinical Specialties, Faculty of Dentistry, Complutense University of Madrid, Madrid, Spain

## Abstract

**Background:**

The present systematic review aims to investigate the guidelines for prescribing Preventive Antibiotic Therapy (PAT) in the placement of dental implants (DIs) without anatomical constraints in healthy patients by clinicians in Europe and to compare them with current recommendations.

**Material and Methods:**

A search was performed in 4 databases: Medline (via PubMed), Web of Science, Scopus, and LILACS. The criteria employed were those described in the PRISMA (Preferred Reporting Items for Systematic Reviews and Meta-Analysis) declaration (PROSPERO Registration number: CRD42022382278).

**Results:**

The electronic search identified 10 studies published between 2010 and 2023 that met the established criteria. Overall, 60.8% ± 24.1% of European professionals routinely prescribe PAT, with the most frequent regimen being perioperative (mean= 46.7% ± 24.3%), followed by postoperative PAT only (mean= 20.3% ± 9.7%).

**Conclusions:**

The most commonly prescribed antibiotic both pre- and postoperatively is amoxicillin and, in allergic patients, clindamycin. In Europe, more doses of PAT are being prescribed than suggested by current recommendations. For this reason, more PAT education is needed in educational curricula to promote a more rational use of antibiotics to reduce the occurrence of antimicrobial resistance.

** Key words:**Antimicrobial stewardship, surveys and questionnaires, antibiotic prophylaxis, dental implants, dental implantation, dental implantation, endosseous, surgical wound infection.

## Introduction

Dental implants (DIs) have become the first choice in the rehabilitation of partially or edentulous patients in recent decades, due to their high predictability, with success rates described at around 98.90% and 94% at 5 and 15 years, respectively (1). As a consequence, the placement of DIs has increased exponentially, from 100,000 - 300,000 DIs per year worldwide in 1988 (2), while in the USA alone, 3 million people are carrying DIs, a number that is growing by 500,000 people each year, according to recent data. Since its invention in the early 1980s (3), treatments with DIs have been accompanied by the prescription of preventive antibiotics (ATB) as DI surgery was considered a procedure with a high risk of infection (4). The reason for this is the presence in the oral cavity of more than 500 - 700 bacterial species, in addition to other non-culturable microorganisms discovered by molecular biological techniques that can contribute to the development of postoperative infections (5,6). This preventive prescription used to be called "antibiotic prophylaxis", however, recently the term "preventive antibiotic therapy" (PAT) was introduced to refer to the administration of ATB in healthy patients to avoid early DI failure and/or the development of infectious complications (7). Therefore, with the spread of DI treatments, the administration of associated PAT has increased. In recent years, much controversy has arisen because certain indices such as NNT ("number needed to treat") - which refers to the number of individuals who must be treated to prevent an adverse event, compared to the expected outcomes in the control group - have shown limited benefit (4,8). In this regard, the NNT for preventing early DI failure has been estimated at 24 (9) to 55 and for preventing the occurrence of postoperative infections at 143 (at the patient level). Specifically, the NNT for preoperative PAT of amoxicillin is 100 and for postoperative PAT of 143 (10). These data are of particular importance in the current era, where antimicrobial resistance is an urgent threat to Public Health. The problem is that an increasing number of infections are becoming more difficult to treat due to the loss of efficacy of these drugs, being associated with an estimated 5 million deaths globally by 2019 (11). An important aspect to consider is the crucial role that dentists play in this problem, as we prescribe 10% of all ATB dispensed worldwide, of which 66% could be avoided as they are not clinically indicated (12). Therefore, several surveys have been published worldwide to identify antibiotic prescribing patterns and frequency of antibiotic prescribing concerning DI treatments to determine whether there is any kind of consensus among practitioners.

In recent years, different antimicrobial stewardship strategies have been implemented. In this regard, the Spanish Society of Implants (13) (SEI - Sociedad Española de Implantes) published in 2022 the first clinical practice guideline (CPG) worldwide on how to prescribe PAT in different DI procedures, such as sinus lifts, immediate DIs, bone augmentations and DI prosthetic phase. Specifically, this CPG recommends the administration of 2 - 3 g amoxicillin 1 h before the placement of DIs without anatomical constraints, i.e., without the need to perform regenerative procedures simultaneously with the placement of DIs, in healthy patients (Grade of recommendation A, GRADE [Grading of Recommendations Assessment, Development and Evaluation] (14) system) although its non-prescription in certain cases could not be considered a wrong approach either (Grade of recommendation B). Therefore, this systematic review aims to investigate the prescribing guidelines for PAT in the placement of DIs without anatomical constraints in healthy patients by professionals dedicated to Oral Implantology in Europe and, secondarily, to determine their suitability based on the current recommendations of the SEI.

## Material and Methods

- Protocol and registration

The present systematic review is reported according to the Preferred Reporting Items for Systematic Reviews and Meta-Analysis (PRISMA) Statement (15), and its protocol was registered on PROSPERO (Registration number: CRD42022382278).

- Focused question

The initial hypothesis is that PAT is being prescribed inappropriately in Europe, with more ATB doses being administered than recommended.

The study aimed to answer the following PICOS ([P] = patient; [I] = intervention; [C] = comparison; [O] = outcome; [S] = study design) question: “In healthy patients of Europe (P) in whom DIs are to be placed without anatomical constraints (I) is PAT (O) being adequately prescribed compared to current recommendations (C) according to survey-based studies (S)?

- Search strategy and database screening

A comprehensive search of the literature was conducted in the following databases: Medline (via PubMed), Web of Science, Scopus and LILACS. The search was performed by two independent researchers (AOSP and NK). MeSH (Medical Subject Headings) terms, keywords and other free terms were used with Boolean operators (OR, AND) to combine searches adapted to each database (Table 1). Mendeley reference software program (Mendeley Reference Manager 2.80.1, Elsevier) was used to automatically discard duplicates, which were verified manually in the resulting list.

**Table 1:**
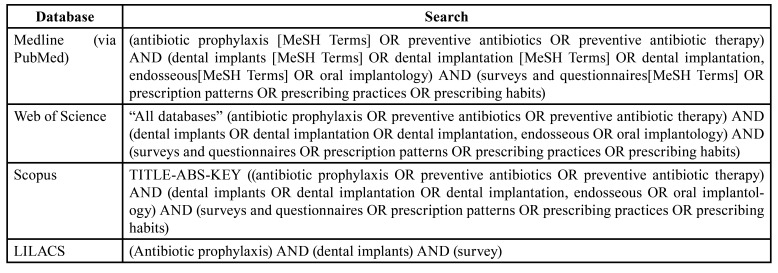
Searches carried out in each of the databases.

- Clinical relevance

Coordinated measures to prevent the spread of antimicrobial resistance have been implemented at the European level in recent decades, however, they do not always translate effectively into rational ATB prescribing. Therefore, it is relevant to know how PAT is being prescribed in DI procedures to establish the adherence of professionals to current recommendations.

- Eligibility criteria

Inclusion criteria: (a) articles published in English; (b) surveys on the prescription of PAT in the placement of DIs in healthy patients without anatomical constraints; and (c) surveys conducted in countries of the European continent. (d) The search was temporarily restricted from 1 January 2010 to 11 November 2023.

Exclusion criteria: (a) Systematic reviews and (b) meta-analysis; (c) prospective and retrospective clinical studies; (d) duplicate articles; (e) books; (f) letters to the Editor; (g) commentaries; (h) case reports; (i) narrative literature reviews; (j) articles studying the use of ATBs in surgical procedures not related to the placement of DIs; and (k) studies investigating the use of ATBs for therapeutic purposes.

- Studies screening and inclusion

Two researchers (AOSP and NK) independently compared their results to ensure completeness and removed duplicates. The full titles and abstracts of the remaining papers were then screened individually. Finally, the full-text articles to be included were selected according to the criteria described above. Disagreements on eligible studies to be included were discussed with a third author (MVMM), and a consensus was reached. The reference list of the included studies was also reviewed to identify other studies potentially meriting inclusion. Agreement between reviewers was measured with the kappa coefficient. The results were also expressed as the concordance between reviewers (%).

- Data extraction

Similarly, data extraction was undertaken by two reviewers (AOSP and NK) independently and results were compared and merged. In case of discrepancy, articles were re-screened by a third reviewer (MVMM). If necessary, study authors were contacted for clarification of missing information.

- Quality assessment and risk of bias

Two independent reviewers (AOSP and MPS) evaluated the methodological quality of eligible studies following the Joanna Briggs Institute (JBI) Critical Appraisal Tool (16). The checklist comprised 10 domains, each being scored as “Yes”, “Unclear” or “No”. The studies were classified as low-quality assessment studies (0 - 5 domains), or as high-quality assessment studies (6 - 10 domains).

## Results

- Study selection

**Figure 1:**
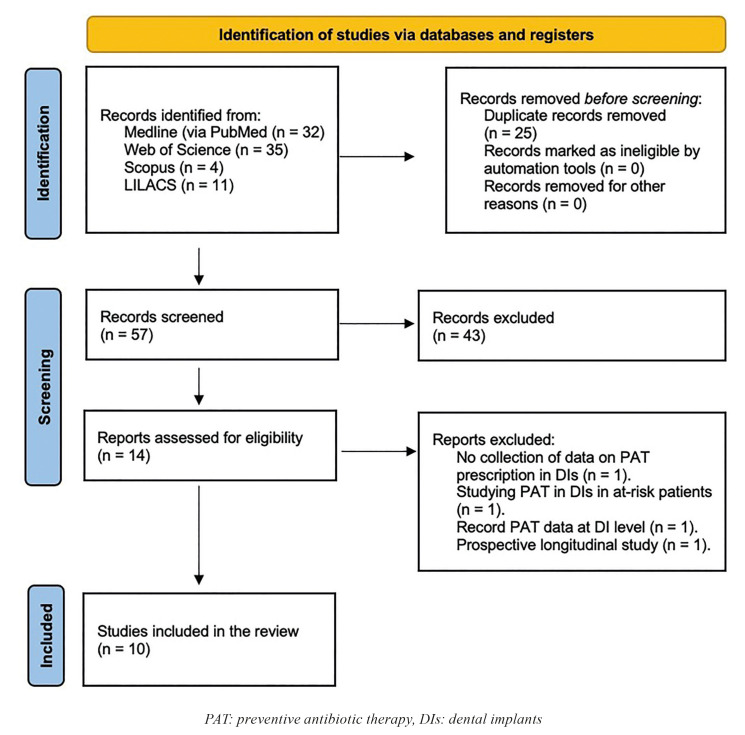
PRISMA flow diagram of the search processes and results.

The search strategy resulted in 82 results (PubMed = 32, Web of Science = 35, Scopus = 4; LILACS = 11), of which 57 remained after removing duplicates. Then, two independent researchers (AOSP and NK) reviewed all the titles and abstracts and excluded 43 papers that were outside the scope of this review or were conducted outside Europe. Thus, we obtained 14 potential references. After reading the full texts of those 14 papers, 4 were discarded for being a survey that did not collect PAT prescription data in the placement of DIs (n = 1) (17); for investigating its appropriateness in at-risk patients (n = 1) (18); for recording data at the DI level (n = 1) (19); and for being a prospective longitudinal study (n = 1) (20). Therefore, 10 studies were included in this systematic review (21-30) (Fig. 1). There was a 90.8% concordance between the two authors (AOSP and NK), with a kappa coefficient of 0.600 (standard error [SE]= 0.009; 95% Confidence Interval [CI], 0.419 - 0.781) for titles and abstracts, and 95% concordance with a kappa coefficient of 0.898 (SE = 0.099; 95%CI, 0.703 - 1.093) for full-text articles, respectively. Thus, the concordance was deemed to be good for titles and abstracts and very good for full-text articles.

- Study characteristics

All surveys were anonymous, except the one carried out by Camacho-Alonso *et al* (26) since of the 200 respondents, 115 answered face-to-face to the interviewer, 35 via telephone and 50 via email. The main objective of the various studies was to determine how common is the prescription of PAT in the treatment of DIs and the dosages used in patients without local or systemic conditions. The methodological design of the study by Khalil *et al* (29) differed slightly from other research as it compared PAT prescribing habits between two surveys of the same sample of Swedish dentists in 2008 and 2012 to determine their adherence to the recommendations of the Swedish Strategic Programme Against Antibiotic Resistance (STRAMA) and the scientific committee of the Health Technology Assessment. We decided to consider the 2012 data as only surveys published from 2010 onwards were included. In addition, 3 studies further investigated: (i) the prescription habits of PAT in certain DI procedures in healthy patients (n = 2) (21,26), (ii) its use to treat early failures or complications in DIs (27) and, in addition, (iii) prescription habits of analgesics and anti-inflammatory drugs (26).

Most surveys were conducted in Spain (21,26-28). They were also conducted in the Netherlands (24), Italy (25) Sweden (29), Turkey (22,23,30), and the UK (23,30). The surveys conducted by the 10 studies were based on those previously published by Deeb *et al* (31) (2015) (n = 3), (24,25,28) by Abukaraky *et al* (26,32) (2011) (n = 1) (26), or by a combination of both (n = 1) (21). Five studies (22,23,27,29,30) did not specify this. Surveys were sent to a total of 25,303 respondents, with response rates ranging from 2.2 (22) to 95.2% (26) (mean = 39.2% ± 31.7%). Two studies did not provide this data (23,30). Therefore, a total of 2,259 respondents were included (Table 2).

**Table 2:**
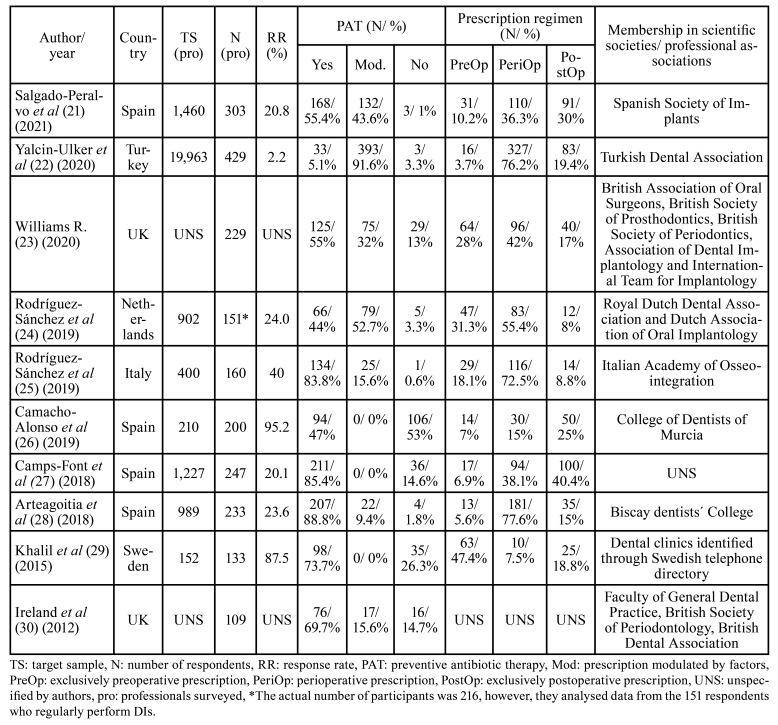
Characteristics of the included studies.

- Professional characteristics

Respondents belonged to various scientific societies and/ or professional associations. In Spain: to SEI (21), College of Dentists of Murcia (26,28) and Biscay dentists' College (28). In Turkey to the Turkish Dental Association (22), in the UK to the British Association of Oral Surgeons, British Society of Prosthodontics, Association of Dental Implantology, International Team for Implantology (23), British Society of Periodontics (23,30), Faculty of General Dental Practice, and British Dental Association (23). In Italy the Italian Academy of Osseointegration (25), in the Netherlands the Royal Dutch Dental Association, and the Dutch Association of Oral Implantology (24), and in Sweden they surveyed dental clinics identified through the Swedish telephone directory (29). Only one survey in Spain did not specify this data (27). The gender distribution of respondents was very heterogeneous, with more men (n = 1,060; 46.9%) than women (n = 495; 21.9%). Two studies did not provide this data (n = 704; 31.2%) (22,27). Age distribution was not possible due to heterogeneity in the collection of this data. Two surveys provided the mean age of respondents (24,26), 4 grouped the age of participants by categories (21,25,28,29), and 4 did not provide this data (22,23,27,30).

- DI procedures in which PAT was studied

Most of the surveys (*n*= 7) (22-25,28-30) asked generically about the type of PAT in DI procedures, so it may refer to the placement of DIs without anatomical constraints in healthy patients. Camacho-Alonso *et al* (26) included a clinical scenario with images. Only two surveys specifically asked about the prescription of PATs in various types of DI procedures (21,26).

- Frequency of PAT prescription

**Figure 2:**
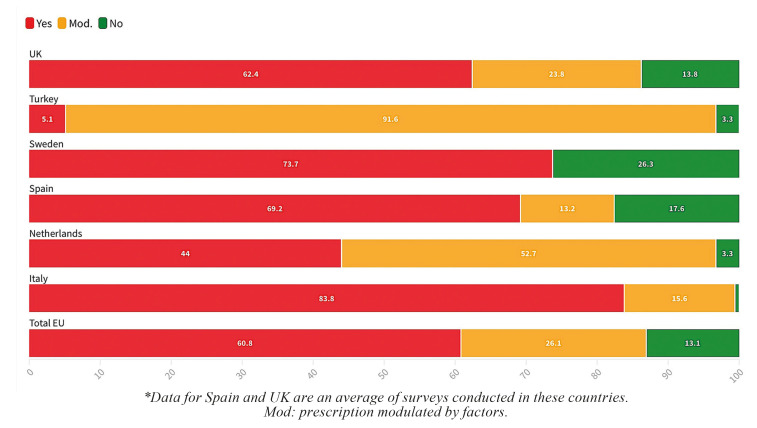
Distribution of the frequency of PAT prescription according to the country.

Overall, the majority of European practitioners prescribe PAT systematically for the placement of DIs (mean: 60.8% ± 24.1%) or occasionally in the presence of certain factors (mean: 26.1% ± 28.1%). Only 13.1% ± 15.5% of respondents do not prescribe PAT in these treatments. The countries that prescribe the most PAT systematically are Italy (83.8%), Sweden (73.7%), Spain (69.2%), and the UK (62.4%), and the least, Turkey (5.1%) and the Netherlands (44%) (Fig. 2).

- Type of PAT prescribed

**Figure 3:**
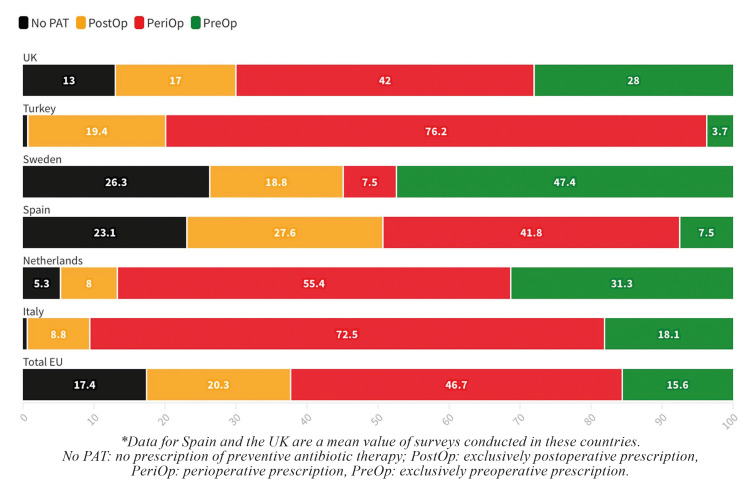
Type of PAT prescribed according to the country.

The overall data show that, in Europe, the most commonly used PAT is perioperative, i.e., before and after surgery (mean: 46.7% ± 24.3%), followed by postoperative PAT (mean: 20.3% ± 9.7%). Preoperative PAT is used in 15.6% ± 14.2%. Sweden is the only country where the most frequent PAT is not perioperative (7.5%) but preoperative (47.4%). The European country that prescribes the most perioperative PAT is Turkey (76.2%), followed by Italy (72.5%). The second most widespread regimen is preoperative in three countries (Netherlands [31.1%], UK [28%], and Italy [18.1%]) and postoperative in two (Spain [mean: 27.6%] and Turkey [19.4%]) (Fig. 3).

Preoperative PAT: type, dose and dosage

A total of 1,288 responses on preoperative PAT guidelines were recorded. Most practitioners start PAT immediately before or 1 h before surgery (n = 708; 54.9%). The most frequently used ATB being amoxicillin (n = 646; 91.2%), namely 2 g (n = 468; 36.3%) or 3 g (n = 135; 10.5%). The second most frequent regimen is PAT initiated 1 or 2 days before surgery (n = 502; 39%), with amoxicillin being the most frequently used ATB (n = 214; 42.6%), namely at a dose of 500 mg/ 3 times/day (TID) (n = 89; 6.9%), followed by amoxicillin/ clavulanic acid (n = 149/ 29.7%) and, more specifically, 875/125 mg/ TID (n = 77; 6%). Indeed, the actual data are higher than reported, as Yalcin-Ulker *et al* (22) recorded the prescription of amoxicillin and amoxicillin/ clavulanic acid together (n = 129; 25.7%), with the most frequent regimen being 1 g, 2 times/day (BID) (n = 124; 9.6%), which would correspond to amoxicillin 1 g per se, and/or amoxicillin/ clavulanic acid 875/125 mg (Supplement 1). The route of choice for all practitioners surveyed was oral. Only one study (22) recorded a limited number of practitioners (n = 9; 2.7%) who administered a PAT of cephalosporins 2 g, 1 h before surgery, via intramuscular (IM) or intravenous (IV).

Postoperative PAT: type, dose and dosage

A total of 1,195 responses were recorded on postoperative PAT guidelines. Most respondents administered PAT for 5 - 7 days (n = 731; 61.2%). The most commonly used ATB is amoxicillin (n = 588; 49.2%) at a dose of 500 mg/ TID, for 5 (n = 97; 8.1%) and 7 days (n = 94; 7.9%). The second most used ATB is amoxicillin/ clavulanic acid (n = 164; 13.7%), at doses of 875/125 mg/ BID, for 6 days (n = 44; 3.7%). The actual data are estimated to be higher than reported, as Yalcin-Ulker *et al* (22) recorded the prescription of amoxicillin and amoxicillin/ clavulanic acid together (n = 379; 31.7%), with the most frequent regimen being 1 g/ BID, for 5 days (n = 215; 18%), which would correspond to amoxicillin 1 g per se, and/or amoxicillin/ clavulanic acid 875/125 mg. On the other hand, prescriptions from two studies conducted in the UK (23,30) and Spain (27,28) could not be recorded as they did not specify all postoperative dosages used (Supplement 2). The route of choice for all practitioners surveyed was oral. Only one study (22) recorded a limited number of practitioners administering cephalosporins 1 g/ BID, for 5 days (n = 1; 0.3%) and clindamycin 300 mg/ BID, for 7 days (n = 1; 0.3%) via IM or IV.

- Antibiotic regimens in case of penicillin allergies

Half of the studies (n = 5) recorded the type of ATB of choice in penicillin-allergic patients (n = 1,001; 44.3%) (21,23,25,26,30). Of these, those carried out in the UK (23,30) and Spain (21,26) mostly used clindamycin. Specifically, in Spain between 58.4% (n = 177) to 100% (n = 200) of respondents prescribed it as their first choice, followed by azithromycin (n = 67; 22.1%) and erythromycin (n = 57; 18.8%) and, to a lesser extent, clarithromycin (n = 2; 0.7%). On the other hand, UK surveys recorded the preferred pre- and postoperative guidelines: in the former, both surveys recorded a higher preference for clindamycin 600 mg, 1 h before surgery (2012 (30): *n* = 45; 43.3%; vs. 2020 (23): *n* = 27; 18%), while 15.4% (n = 16) in 2012 (30) to 14% (*n*= 20) in 2020 (23) did not prescribe ATB in allergic patients preoperatively. Postoperatively, the most commonly used ATB was metronidazole. In 2012 (30) the most commonly used regimen was 200 mg/ TID, for 7 days (n = 10; 10%) and 400 mg/ TID, for 5 days (n = 10; 10%), while 13% did not prescribe ATB (n = 13). In contrast, in 2020 (23) the first choice was metronidazole 400 mg/ TID, for 5 (n = 14; 12%) and 7 days (n = 12; 10%), followed by erythromycin 250 mg/ 4 times/day (QID) (n = 7; 6%) and 500 mg/ QID (n = 7; 6%), both for 5 days. In Italy (25) the most commonly used ATB in allergic patients were macrolides (n = 94; 84%), of which 66% (n = 62) prescribed clarithromycin. Five studies pooling 55.7% of the responses (n = 1,258 participants) did not record the ATB of choice in these patients (22,24,27-29).

- Quality assessment and risk of bias

Using the predetermined 10 domains for the methodological quality assessment according to the JBI Critical Appraisal Tool (16), we determined that 6 of the 10 studies were found to be of high-quality (21,22,24-26,28), while 4 studies were of low-quality (23,27,29,30) (Table 3).

**Table 3:**
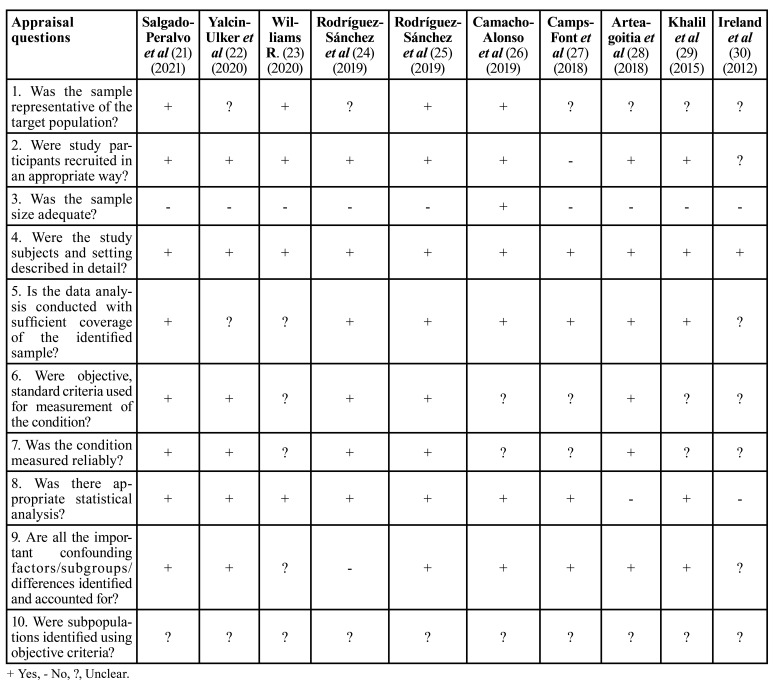
JBI Critical Appraisal Checklist.

## Discussion

The prescription of PAT has become standardised in DI treatments since the first protocols described by Branemark (3) in which phenoxymethylpenicillin was administered 1 h before surgery and for 10 days postoperatively. This is reflected by the fact that 60.8% of the European professionals surveyed carry it out systematically. These data would not be too alarming as it is currently recommended to prescribe 2 or 3 g of amoxicillin 1 h before the placement of DIs without anatomical constraints (13). However, it is worrying that, except in a survey conducted in Sweden (29), in the rest of the European countries the most widespread PAT regimen is perioperative, with the most frequent postoperative duration being 5 to 7 days, which implies a greater amount of ATB doses than recommended. These data are confirmed by a systematic review and meta-analysis published in 2020 (33) which showed that the mean dose of PAT prescribed per DI surgery worldwide is 10,724 mg, which is significantly higher than the suggested 2,000 mg.

These data have to be interpreted in the context that the routine prescription of PAT in healthy patients does not present a justified benefit-risk ratio, as the reduction in the probability of early DI failure is only 2% (4) and PAT is not useful in preventing postoperative infections (10). Furthermore, 93.2% of practitioners prescribe ATB when they do occur (26), significantly increasing the overall duration of ATB treatment. This not only increases the risk of antimicrobial resistance, but also the probability of idiosyncratic and dose-dependent adverse reactions that may compromise the patient's life, it may cause toxicity on various target organs, alterations in the usual bacterial flora of the mucosa, and interactions with other drugs that the patient is already taking. For these reasons, there is much controversy regarding the value of PAT in these cases. It should also be considered that when ATB are prescribed for preventive purposes, they should be administered preoperatively (at least 1 h before surgery) to achieve an adequate blood concentration, sufficient to exceed the minimum inhibitory concentration by 2 to 4 times. Exceeding this therapeutic range creates a window of selection for therapeutic overdose known as the "mutant selection window", which modifies the susceptibility of bacteria to ATB, making them resistant (34). The non-prescription of PAT could also not be considered a wrong approach in healthy, young, non-smoking patients, and in the presence of certain techniques such as, possibly, guided surgery which allows the placement of DIs in a minimally invasive way without elevating mucoperiosteal flaps (35).

Futhermore, the classic choice for PAT in penicillin-allergic individuals is clindamycin. However, in these patients, a sensitivity test should be performed to allow a confirmatory diagnosis, as a recent systematic review (36) found a 3-fold increased risk of early implant failure in patients with self-reported penicillin allergy. It is therefore unknown whether this increased risk is attribuTable to the use of clindamycin, to allergy per se, or a combination of both factors.

Possibly the most effective antimicrobial stewardship measure to modify current PAT prescribing habits is through specialised training in ATB therapy in Oral Implantology. In this regard, a significant reduction in the number of routine PAT prescriptions (29) was achieved in Sweden, as well as in their administration beyond the day of the intervention. This improvement was achieved following the publication by The Swedish Council on Health Technology Assessment (2010) of recommendations and a literature review, respectively, on PAT prescription in these procedures. These findings are in line with those reported by Camacho-Alonso *et al* (26) in Spain, who observed that dentists with more than 10 years of experience who attended courses on ATB and who read scientific articles prescribe significantly less of these drugs. Furthermore, factors related to scientific evidence, such as knowledge acquired during undergraduate university studies, in postgraduate courses or at conferences and congresses, reading scientific material and published guidelines, are the most important factors in decision-making. Therefore, efforts are needed to promote the current recommendations on PAT in DI procedures (13) to reach the maximum number of professionals, such as informative talks, promotion at congresses and scientific meetings, as well as the sending of information leaflets with the indications for PAT summarised so that professionals with a more clinical profile are informed. These data become even more relevant after learning that dentists who read publications are more likely to prescribe a single dose of PAT than those who do not and that a third of them will modify their prescribing patterns (29).

For this systematic review, we took as a reference the review published by Bernabeu-Mira *et al* (37) in 2021. However, these authors included in their research surveys conducted on professionals from all over the world published up to 2020. In the present study, it was decided to focus on current PAT prescribing habits in Europe. Thus, the 6 studies included in the aforementioned article, as well as 4 other publications in the last 3 years, were taken into consideration. Therefore, the main difference is the delimitation of the target population and the restatement and updating of the object of study. In addition, the SEI CPG (13) was subsequently published, making it possible to compare the prescribing habits of professionals in Europe with those currently recommended.

One of the main limitations of the present study was the heterogeneity between the different surveys, which made it difficult to establish a comparison between them. Also, the vast majority of the studies did not ask about the prescription of PAT in various DI procedures, as the same guideline cannot be extrapolated to all indications and, in addition, as they are based on surveys, it is not possible to establish the veracity of the answers provided by the participants. Future studies should be aimed at investigating possible changes in PAT prescribing habits to determine practitioner adherence to current recommendations concerning specific DI procedures.

## Conclusions

 With the limitations of the present study, it can be concluded that PAT is being inappropriately prescribed in DI procedures. Recent recommendations suggest prescribing 2 or 3 g of amoxicillin 1 h before the placement of DIs without anatomical constraints, while the most frequently used regimen in several European countries is perioperative, with the most frequent postoperative duration being 5 to 7 days, which implies a greater amount of ATB doses. The most commonly used pre- and postoperative ATB is amoxicillin and, in allergic patients, clindamycin.
